# Nurses' Pension Funds

**Published:** 1887-02-26

**Authors:** Annie Briggs

**Affiliations:** Nursing Institute, Bangor, N. W.


My nurses are anxious to know whether the "National Nurses' Pension, Sick, and Annuity Fund" which you proposed, has been started. If it is already in existence I shall be so much obliged if you will let me have the particulars of it. If it is not yet started, please do not trouble to answer this, as I shall understand. I must congratulate you on the great success of The Hospital, which I see every week, and find it most interesting and useful. Annie Briggs.

Nursing Institute, Bangor, N. W., Feb. 14th, 1887.

*⁎* It is hoped that the Jubilee Year will not end before a practical scheme for affording nurses an opportunity of joining the National Pension Fund has been formulated. The difficulties are found to lie (1) in the inability of the nurses to pay a sufficient sum to realise £25 per annum pension; (2) in the reluctance of Hospital Committees to increase the cost of nursing by contributing to such a fund; and (3) in finding evidence to prove that any but a very few nurses care in reality whether such a fund be established or not, seeing it is stated that the majority do not remain nurses for more than a few years of their lives under existing conditions. We shall be glad to hear the views of all nurses and hospital officials on this subject.—Ed. T. H.

